# *UMOD* Genotype and Clinical Outcomes in Heart Failure Patients Treated with Loop Diuretics: A UK Biobank Pharmacogenetic Cohort Study

**DOI:** 10.1002/cpt.70426

**Published:** 2026-08-06

**Authors:** Reinhold Kreutz, Pimrapat Gebert, Jingwen Luo, Paraskevi Tassopoulou, Juliane Bolbrinker, Antonios Douros

**Affiliations:** ^1^ Institute of Clinical Pharmacology and Toxicology, Charité ‐ Universitätsmedizin Berlin Berlin Germany; ^2^ Institute of Biometry and Clinical Epidemiology, Charité ‐ Universitätsmedizin Berlin Berlin Germany; ^3^ School of Epidemiology and Public Health, University of Ottawa Ottawa Ontario Canada; ^4^ Department of Epidemiology Biostatistics and Occupational Health, McGill University Montreal Quebec Canada

## Abstract

Uromodulin (UMOD) regulates tubular sodium handling and modulates NKCC2, the molecular target of loop diuretics (LD). Although *UMOD variants* have been associated with blood pressure and hypertension, their pharmacogenetic relevance in heart failure (HF) remains unknown. We assessed whether the *UMOD rs13333226* genotype is associated with clinical outcomes among patients with HF who initiated LD. We assembled a cohort of UK biobank (UKB) participants diagnosed with HF who initiated LD. Patients were followed in an intention‐to‐treat fashion for up to 12 months. Cox models estimated confounder‐adjusted hazard ratios (HRs) and 95% confidence intervals (CIs) comparing *rs13333226‐AA* versus *rs13333226‐AG/GG* genotypes. The primary effectiveness outcome was worsening HF, defined as hospitalization for HF or death with HF as the primary cause. Additional effectiveness and safety outcomes were major adverse cardiovascular events (MACE), acute kidney injury (AKI), and all‐cause mortality. Secondary analyses stratified by age and sex. Sensitivity analyses restricted follow‐up to 3 and 6 months. Among 1,426 HF patients initiating LD (92% furosemide, mean age 68 years, 34% female), the *rs13333226‐AA* genotype was not associated with worsening HF (HR, 0.95; 95% CI, 0.76–1.20), MACE (HR, 0.97; 95% CI, 0.62–1.52), AKI (HR, 1.05; 95% CI, 0.68–1.64), or all‐cause mortality (HR, 1.17; 95% CI, 0.76–1.78). There was no effect modification by demographics. Shorter follow‐up periods did not affect the results. In HF patients initiating LD, *UMOD* rs13333226 was not associated with effectiveness or safety outcomes. These findings do not support a clinically meaningful pharmacogenetic role for this variant.

**Study Highlights**

**WHAT IS THE CURRENT KNOWLEDGE ON THE TOPIC?**

Uromodulin (UMOD) regulates tubular sodium handling and modulates NKCC2, which is the molecular target of loop diuretics. *UMOD variants* have been associated with blood pressure and hypertension.

**WHAT QUESTION DID THIS STUDY ADDRESS?**

Leveraging the linkage between the UK Biobank and prescription data that was recently made available, we addressed whether there is an association between the *UMOD rs13333226* genotype and the risk of adverse clinical outcomes among patients with heart failure who initiated treatment with loop diuretics.

**WHAT DOES THIS STUDY ADD TO OUR KNOWLEDGE?**

In a cohort of > 1400 patients with heart failure who initiated treatment with loop diuretics, the *UMOD rs13333226* genotype was not associated with the risks of worsening heart failure, major adverse cardiovascular events, acute kidney injury, or all‐cause mortality. There was no major effect measure modification by age, sex, or chronic kidney disease.

**HOW MIGHT THIS CHANGE CLINICAL PHARMACOLOGY OR TRANSLATIONAL SCIENCE?**

While neutral, these findings provide important evidence regarding the clinical relevance of a widely studied renal sodium transport pathway and help refine expectations for pharmacogenetic approaches to diuretic therapy in heart failure.

Loop diuretics are frequently used in cardiovascular medicine and particularly in the symptomatic management of fluid overload among patients with heart failure (HF).^1^, ^2^, ^3^, ^4^ Their mechanism of action is based on blocking the Na^+^‐K^+^‐2Cl^−^ cotransporter (NKCC2) in the nephron; this results in the inhibition of sodium reabsorption with increased excretion of sodium, chloride, and water, as well as volume depletion and decongestion.^3^ Of note, loop diuretics show considerable variability in their treatment effects. For example, the associated reduction in blood pressure (BP) may vary by several mmHg between individuals.^5^ In patients with HF, considerable interindividual differences in diuretic response have also been demonstrated, reflected by variability in natriuresis, diuresis, weight loss, and decongestion following loop diuretic therapy, with poorer diuretic response being associated with adverse clinical outcomes.^3^, ^6^, ^7^ This variability remains largely unexplained, with potential contributing factors including differences in adherence, dietary sodium intake, or renal function, and concurrent medication use. Importantly, although recent studies have suggested that genetic factors may also influence the effects of diuretics, respective data on loop diuretics are limited.^8^

A genetic factor that could potentially impact the effectiveness and safety of loop diuretics among patients with HF is the *UMOD* gene.^9^, ^10^, ^11^ UMOD encodes uromodulin, a protein expressed primarily in the thick ascending limb of the loop of Henle, which directly modulates NKCC2 function and sodium handling.^9^, ^10^, ^11^ Genetic variation within *UMOD*, particularly the rs13333226 single nucleotide polymorphism (SNP), can influence uromodulin expression levels, renal sodium reabsorption, and BP regulation. The rs13333226 SNP is a common intronic variant (minor allele frequency ~ 18% in European populations) located upstream of *UMOD* that tags a regulatory haplotype influencing UMOD expression.^10^ Indeed, individuals carrying the rs13333226‐AA genotype exhibit higher UMOD expression, increased sodium retention, and elevated BP compared to AG/GG genotype carriers.^9^

Importantly, a recent genotype‐blinded trial in uncontrolled hypertension showed that carriers of the *UMOD* rs13333226‐AA genotype had a modestly greater ambulatory systolic BP (SBP) reduction with a loop diuretic (torsemide) when compared with AG/GG carriers.^8^ Although the between‐genotype difference was small and limited to BP outcomes, the findings support a biologically plausible UMOD‐NKCC2 interaction and suggest that genetic variation may influence loop diuretic response. Because loop diuretics are not first‐line therapy in patients with uncomplicated hypertension and preserved glomerular filtration rate^12^, ^13^, ^14^ but are central to HF management, HF represents a clinically relevant setting in which to test this pharmacogenetic hypothesis.

Considering the above, a hypothesis‐testing study assessing whether genetic variation within *UMOD* can impact the clinical effects of loop diuretics in the management of fluid overload among patients with HF would help address an important knowledge gap. To this end, we conducted a large pharmacogenetic cohort study assessing whether the *UMOD* rs13333226 genotype is associated with the risk of adverse clinical outcomes among patients with HF who initiate loop diuretics.

## METHODS

### Data source

We used the United Kingdom (UK) Biobank (UKB) linked to electronic medical records from general practitioners, hospitalization records, and vital statistics data.^15^ The UKB is a large‐scale, prospective, population‐based cohort study that enrolled approximately 500,000 participants in the UK between 2006 and 2010. The UKB conducted an extensive phenotype assessment of participants at baseline, including demographic characteristics, anthropometric variables (e.g., body mass index [BMI]), clinical measurements (e.g., BP), laboratory test results (e.g., estimated glomerular filtration rate [eGFR]), lifestyle variables (e.g., smoking habits), comorbidities, and medication use. Furthermore, the UKB provides extensive genetic information including large‐scale exome and whole‐genome sequencing.^15^

Electronic medical records from general practitioners contain longitudinal information on anthropometric, clinical, and lifestyle variables, laboratory test results, outpatient medical diagnoses (coded using Read codes versions 2 and 3), and outpatient medical procedures. Moreover, they contain detailed data on drug prescriptions issued by general practitioners, including compound name (coded using British National Formulary codes, Read codes, and Dictionary of Medicines and Devices), date of prescription, and quantity prescribed. Linkage to electronic medical records is currently available for 230,000 participants of the UKB (https://www.ukbiobank.ac.uk/). Hospitalization records contain admission and discharge diagnoses (coded using the International Classification of Diseases, 10th Revision [ICD‐10]), and inpatient medical procedures (coded using the Office of Population Censuses and Surveys Classification of Surgical Operations and Procedures, Version 4). Finally, vital statistics data contain the date and the underlying cause of death (coded using ICD‐10). The study protocol was approved by the data custodians (Application ID: 568509).

### Study cohort

Out of the source population of the UKB, we identified participants with linked electronic medical records and a diagnosis of HF. HF was defined based on a combination of inpatient diagnostic codes, outpatient diagnostic codes, and self‐reported information that was obtained upon UKB enrollment (**Tables** **S1** and **S2**). Of those, we assembled a study cohort including patients who initiated treatment with any loop diuretic approved in the UK (i.e., furosemide, torsemide, bumetanide) after the diagnosis of HF and after their enrollment in the UKB. The date of treatment initiation with a loop diuretic defined the date of cohort entry. We excluded UKB participants with a prescription for a loop diuretic in the 6 months prior to focus on patients initiating treatment. We also excluded UKB participants with less than 365 days of medical history in the electronic medical records (to assess prevalent use of loop diuretics and to measure comorbidities and comedications) and without genotype data. Patients were followed from study cohort entry until the earliest of the following: the occurrence of one of the study outcomes (defined below), death (for non‐mortality outcomes), 12 months after cohort entry, or the end of UKB data availability.

### Exposure definition

We used an intention‐to‐treat exposure definition with a maximum follow‐up duration of 12 months. By design, the two exposure groups included (i) initiators of loop diuretics with the rs13333226‐AA genotype, and (ii) initiators of loop diuretics with the rs13333226‐AG or rs13333226‐GG genotypes. Accordingly, rs13333226 was analyzed under a recessive genetic model (AA versus AG/GG). This is consistent with prior functional evidence that has linked the AA genotype to higher expression of uromodulin and altered sodium handling.^9^ Genotype data were accessed from the UKB March 2018 imputed dataset (GRCh37 alignment).

### Outcome definition

The primary effectiveness outcome was worsening of HF, defined as either hospitalization with HF (ICD‐10 codes I11.0, I13.0, I13.2, I50.x; anywhere in the hospitalization record) or death with HF as the primary cause. The secondary effectiveness outcome was major adverse cardiovascular events (MACE), defined as a composite of myocardial infarction (MI), stroke, and cardiovascular death. MI was defined as hospitalization with MI (ICD‐10 codes I21.x, anywhere in the hospitalization record), stroke was defined as hospitalization with stroke (ICD‐10 codes I60.x, I61.x, I62.x, I63.x, and I64.x, anywhere in the hospitalization record), and cardiovascular death was defined as death with cardiovascular cause (ICD‐10 codes I00.x‐I77.x [except I46.9]) as primary cause. The primary safety outcome was acute kidney injury (AKI), defined as a hospitalization with AKI (ICD‐10 code N17x, anywhere in the hospitalization record) or death with AKI as the primary cause. The secondary safety outcome was all‐cause mortality.

### Covariates

Given that all patients in the study cohort were initiators of loop diuretics who were previously diagnosed with HF, we expected confounding to be limited. However, we did consider the following potential confounders in our statistical analyses: age (modeled flexibly using restricted cubic splines with 4 interior knots), sex, BMI category (underweight [< 18.5 kg/m^2^], normal weight [18.5–24.9 kg/m^2^], overweight [25.0–29.9 kg/m^2^], obese [≥ 30.0 kg/m^2^] or unknown; last measurement before cohort entry), and smoking status (current, past, never).

We also descriptively assessed BP levels (SBP < 140 mmHg and diastolic BP [DBP] < 90 mmHg, SBP ≥ 140 mmHg or DBP ≥ 90 mmHg, or unknown) and eGFR using the last measurement before cohort entry. We also descriptively assessed the following comorbidities: arterial hypertension, prior MI, prior stroke, chronic kidney disease (CKD), diabetes mellitus, depression, liver disease (all measured based on outpatient or inpatient diagnostic codes ever before cohort entry), and cancer (measured based on outpatient or inpatient diagnostic codes in the year before cohort entry). Finally, we descriptively assessed the use of the following comedications: statins, beta‐blockers, angiotensin‐converting enzyme (ACE) inhibitors, angiotensin‐receptor blockers (ARBs), mineralocorticoid receptor antagonists (MRAs), sodium‐glucose cotransporter‐2 (SGLT2) inhibitors, thiazide‐ and thiazide‐like diuretics, non‐steroidal anti‐inflammatory drugs, antiplatelet agents, and oral anticoagulants (all measured in the year before cohort entry).

### Statistical analyses

Covariate balance between exposure groups was assessed via standardized mean differences (SMDs). We used Cox proportional hazards models to estimate adjusted hazard ratios (HRs) and 95% confidence intervals (CIs) of the study outcomes associated with use of loop diuretics among patients with HF and the AA genotype versus use of loop diuretics among patients with HF and the AG or GG genotypes. Moreover, we conducted secondary analyses to assess potential effect measure modification stratifying by age (< 65 years versus ≥ 65 years), sex assigned at birth, and CKD. Finally, we conducted sensitivity analyses to address different potential sources of bias. First, we restricted the analyses to individuals of European ancestry. Second, we applied shorter maximum follow‐up periods of 3 months and 6 months. Third, we imposed stricter outcome definitions for worsening of HF, MACE, and AKI by including only diagnoses in the primary position in the hospitalization records. All statistical analyses were performed within the secure UKB Trusted Research Environment based on de‐identified data using R version 4.5.2 (R Foundation for Statistical Computing, Vienna, Austria).

## RESULTS

**Figure 1:**
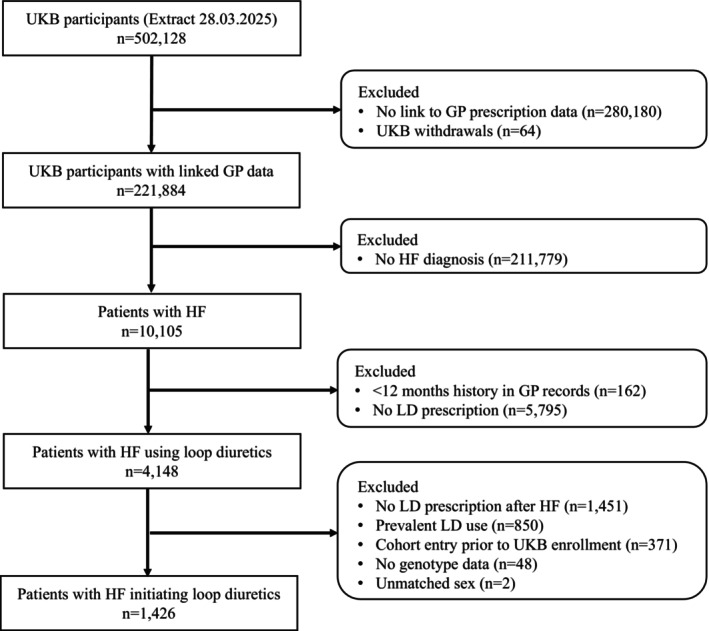
Flowchart illustrating the construction of the study cohort. GP, general practitioner; HF, heart failure; LD, loop diuretics; UKB, United Kingdom Biobank.

Overall, our study cohort included 1,426 UKB participants who were diagnosed with HF and then initiated treatment with loop diuretics (**Figure** **1**). The most common loop diuretic in the study cohort was furosemide (93%). The majority of patients had the AA genotype (*n* = 948), followed by the AG genotype (*n* = 425), and the GG genotype (*n* = 53). Mean age was 68 years, and 34% of patients were female. The prevalence of comorbidities and the utilization patterns of comedications were typical for a population of HF patients. As expected, clinical characteristics including the use of medications recommended in the management of HF were well‐balanced between exposure groups, with all descriptively assessed covariates except for eGFR showing SMDs < 10% (**Table** **1**). The difference in mean eGFR, albeit yielding an SMD > 10%, was small (77 mL/min per 1.73m^2^ versus 80 mL/min per 1.73m^2^) and was not deemed clinically significant.

**Table 1:** Clinical characteristics of study cohort members stratified by *UMOD* genotype

	AA (*n* = 948)	AG/GG^a^ (*n* = 478)	Total (*n* = 1426)	SMD
Demographic characteristics				
Age in years, mean (SD)	67.6 (6.0)	67.6 (6.6)	67.6 (6.2)	0.006
Female sex	321 (33.9%)	165 (34.5%)	486 (34.1%)	−0.007
European ancestry only	819 (86.4%)	406 (84.9%)	1,225 (85.9%)	0.015
Clinical and lifestyle variables				
BMI in kg/m^2^, mean (SD)	29.9 (5.8)	29.8 (5.6)	29.9 (5.7)	0.031
< 18.5	7 (0.7%)	3 (0.6%)	10 (0.7%)	
18.5–24.9	166 (17.5%)	95 (19.9%)	261 (18.3%)	
25.0–29.9	334 (35.2%)	144 (30.1%)	478 (33.5%)	
≥ 30.0	438 (46.2%)	233 (48.7%)	671 (47.1%)	
Missing	3 (0.3%)	3 (0.6%)	6 (0.4%)	
Smoking status				0.014
Ex‐smoker	498 (52.5%)	239 (50.0%)	737 (51.7%)	
Non‐smoker	344 (36.3%)	192 (40.2%)	536 (37.6%)	
Smoker	106 (11.2%)	47 (9.8%)	153 (10.7%)	
Blood pressure levels				0.011
SBP < 140 mmHg & DBP < 90 mmHg	638 (67.3%)	325 (68.0%)	963 (67.5%)	
SBP ≥ 140 mmHg or DBP ≥ 90 mmHg	295 (31.1%)	143 (29.9%)	438 (30.7%)	
Missing	15 (1.6%)	10 (2.1%)	25 (1.8%)	
eGFR in mL/min per 1.73 m^2^, mean (SD)	76.6 (15.7)	79.9 (15.2)	77.7 (15.6)	−0.212
Missing	49 (5.2%)	25 (5.2%)	74 (5.2%)	
Comorbidities				
Arterial hypertension	727 (76.7%)	353 (73.8%)	1,080 (75.7%)	0.028
Prior MI	358 (37.8%)	173 (36.2%)	531 (37.2%)	0.016
Prior stroke	139 (14.7%)	79 (16.5%)	218 (15.3%)	−0.019
Chronic kidney disease	270 (28.5%)	100 (20.9%)	370 (25.9%)	0.076
Diabetes mellitus	276 (29.1%)	147 (30.8%)	423 (29.7%)	−0.016
Depression	209 (22.0%)	96 (20.1%)	305 (21.4%)	0.020
Cancer	145 (15.3%)	70 (14.6%)	215 (15.1%)	0.007
Liver disease	68 (7.2%)	25 (5.2%)	93 (6.5%)	0.019
Comedications				
Statins	656 (69.2%)	309 (64.6%)	965 (67.7%)	0.046
Beta‐blockers	674 (71.1%)	333 (69.7%)	1,007 (70.6%)	0.014
ACE inhibitors	589 (62.1%)	293 (61.3%)	882 (61.9%)	0.008
Angiotensin‐receptor blockers	213 (22.5%)	108 (22.6%)	321 (22.5%)	−0.001
Mineralocorticoid receptor antagonists	180 (19.0%)	84 (17.6%)	264 (18.5%)	0.014
SGLT2 inhibitors	2 (0.2%)	0 (0%)	2 (0.1%)	0.002
Thiazide‐ and thiazide‐like diuretics	153 (16.1%)	55 (11.5%)	208 (14.6%)	0.046
Non‐steroidal anti‐inflammatory drugs	277 (29.2%)	142 (29.7%)	419 (29.4%)	−0.005
Antiplatelet agents	518 (54.6%)	237 (49.6%)	755 (52.9%)	0.051
Oral anticoagulants	360 (38.0%)	167 (34.9%)	527 (37.0%)	0.030
Loop diuretics				−0.009
Bumetanide	74 (7.8%)	33 (6.9%)	107 (7.5%)	
Furosemide	874 (92.2%)	445 (93.1%)	1,319 (92.5%)	

Abbreviations: ACE, angiotensin‐converting enzyme; BMI, body mass index; BP, blood pressure; DBP, diastolic blood pressure; eGFR, estimated glomerular filtration rate; MI, myocardial infarction; SBP, systolic blood pressure; SD, standard deviation; SGLT2, sodium‐glucose cotransporter‐2; SMD, standardized mean difference.

^a^ 53 of 1,426 participants (3.7%) had GG *UMOD* genotype.

**Figure 2:**
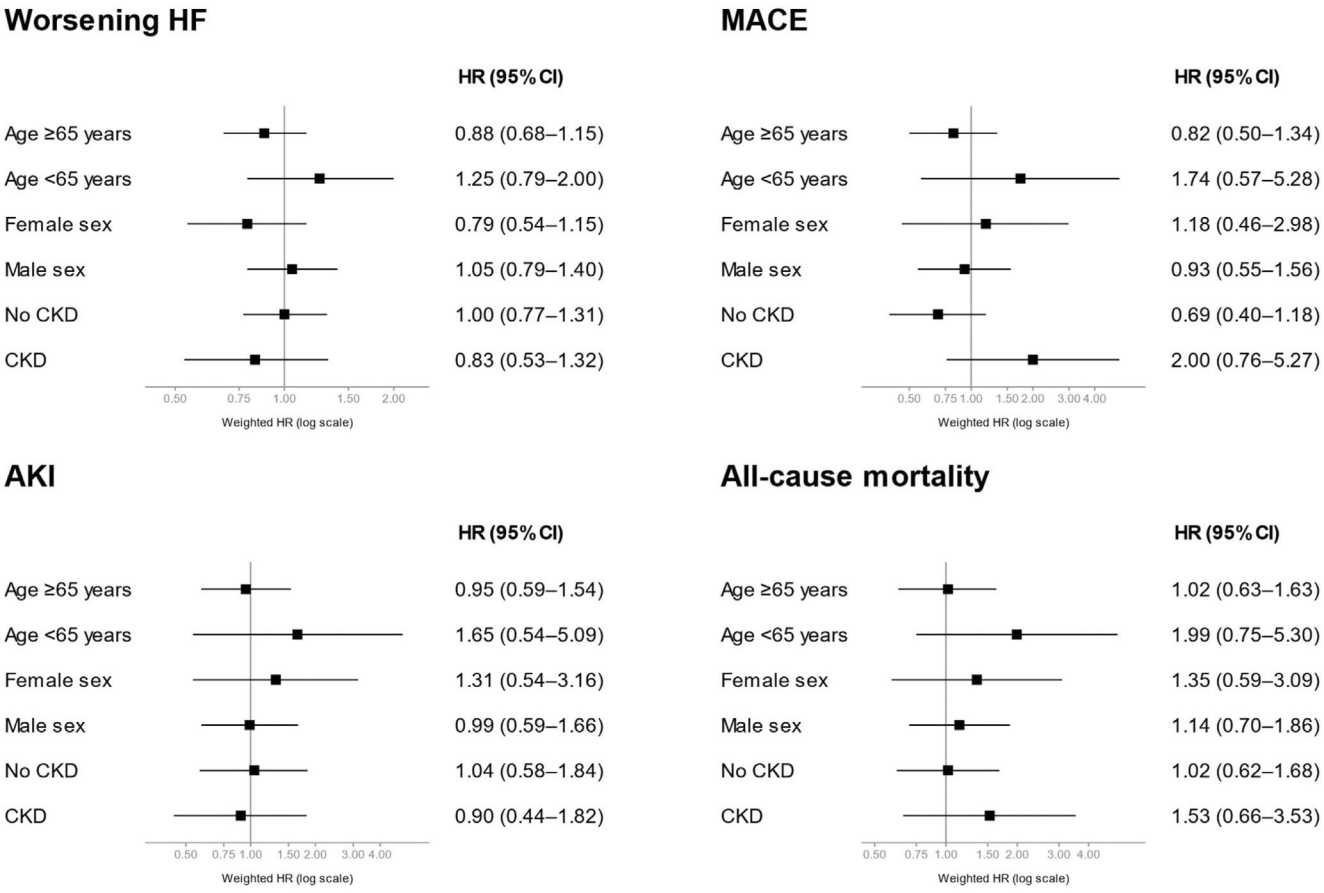
Forest plot summarizing the findings of the secondary analyses. AKI, acute kidney injury; CI, confidence interval; CKD, chronic kidney disease; HF, heart failure; HR, hazard ratio; MACE, major adverse cardiovascular events.

Among patients with HF who initiated loop diuretics, the AA genotype was not associated with the risk of worsening of HF (HR, 0.95; 95% CI, 0.76 to 1.20), MACE (HR, 0.97; 95% CI, 0.62 to 1.52), AKI (HR, 1.05; 95% CI, 0.68 to 1.64), or all‐cause mortality (HR, 1.17; 95% CI, 0.76 to 1.78), when compared to the rs13333226‐AG/GG genotypes (**Table** **2**). Moreover, there was no effect measure modification by age or sex. However, there was a potential effect measure modification by CKD for the outcome MACE (no CKD: HR, 0.69; 95% CI, 0.40 to 1.18/CKD: HR, 2.00; 95% CI, 0.76 to 5.27), albeit the analyses were not conclusive given limited statistical power (results of all secondary analyses are summarized in **Figure** **2** and shown in detail in **Tables** **S3** **–** **S6**). Finally, sensitivity analyses addressing different potential sources of bias yielded findings that were overall consistent with those in the primary analyses (**Tables** **S7** **–** **S10**).

**Table 2:** Effectiveness and safety of the AA *UMOD* genotype among patients with HF initiating loop diuretics

	*N* Patients	*N* Events	*N* Person‐years	IR (95% CI) per 100 person‐years	Crude HR (95% CI)	Adjusted HR^a^ (95% CI)
Worsening HF						
AA	948	218	781	27.9 (24.3–31.6)	0.97 (0.78–1.22)	0.95 (0.76–1.20)
AG/GG	478	113	395	28.6 (23.5–33.9)	1.00 (reference)	1.00 (reference)
MACE						
AA	948	57	889	6.4 (4.8–8.1)	0.99 (0.63–1.55)	0.97 (0.62–1.52)
AG/GG	478	29	448	6.5 (4.2–8.9)	1.00 (reference)	1.00 (reference)
AKI						
AA	948	62	882	7.0 (5.3–8.8)	1.07 (0.69–1.67)	1.05 (0.68–1.64)
AG/GG	478	29	443	6.5 (4.3–9.0)	1.00 (reference)	1.00 (reference)
All‐cause mortality						
AA	948	73	904	8.1 (6.3–10.0)	1.19 (0.78–1.82)	1.17 (0.76–1.78)
AG/GG	478	31	459	6.8 (4.6–9.1)	1.00 (reference)	1.00 (reference)

Abbreviations: AKI, acute kidney injury; BMI, body mass index; CI, confidence interval; HF, heart failure; HR, hazard ratio; IR, incidence rate; MACE, major adverse cardiovascular events; UMOD, uromodulin.

^a^ Adjusted for age, sex, BMI, and smoking status. Participants with missing BMI (*n* = 6) were suppressed.

## DISCUSSION

Contemporary European^1^ and US American^2^ HF guidelines position loop diuretics as foundational therapy for the relief of congestion across all ejection fraction phenotypes and as first‐line treatment for decongestion in acute or worsening HF.^4^, ^16^ They carry a Class I recommendation for reducing signs and symptoms of fluid overload.^1^, ^2^ In contrast to disease‐modifying therapies such as ACE inhibitors, ARBs, angiotensin receptor‐neprilysin inhibitors, beta‐blockers, MRAs, or SGLT2 inhibitors, loop diuretics have no proven mortality benefit in randomized trials and are primarily used to achieve and maintain euvolemia.^1^, ^2^, ^4^ Nevertheless, congestion is a dominant driver of symptoms, impaired quality of life, and HF hospitalizations, and worsening HF events are strongly associated with adverse prognosis and costs.^4^, ^16^, ^17^, ^18^, ^19^ Therefore, optimizing diuretic response remains clinically important.

Precision medicine in HF has largely focused on disease‐modifying therapies, whereas pharmacogenetic approaches to symptomatic treatments remain largely underexplored. UMOD is selectively expressed in the thick ascending limb and has been implicated in sodium handling and regulation of NKCC2 activity, the pharmacologic target of loop diuretics, supporting a biologically plausible genotype‐drug interaction.^8^, ^9^, ^10^, ^11^ This provides a clear rationale to evaluate whether *UMOD* variation modifies the effectiveness and safety of loop diuretics in HF, a setting in which diuretic exposure is frequent and clinically highly relevant.

A recent randomized trial among patients with uncontrolled hypertension suggested a plausible interaction between *UMOD* rs13333226 and NKCC2, raising the possibility of genotype‐guided loop diuretic therapy.^8^ This provided supportive human evidence motivating evaluation of this pharmacogenetic hypothesis in HF, where loop diuretics play a central therapeutic role. However, in our large pharmacogenetic cohort of more than 1,400 patients with HF initiating loop diuretics, rs13333226 was not associated with the risks of worsening HF, MACE, AKI, or all‐cause mortality. Secondary analyses showed no major effect measure modification by demographic factors or chronic kidney disease, and results were consistent in sensitivity analyses restricted to shorter follow‐up periods.

Our study has strengths. First, it is the first to assess the clinical impact of the *UMOD* genotype on loop diuretic outcomes among patients with HF, addressing a clinically relevant and mechanistically grounded question. Second, the large cohort enabled relatively precise effect estimates and multiple secondary analyses exploring the existence of potential effect measure modifiers. That being said, additional statistical power would have been necessary to exclude small‐to‐moderate effects. Third, by excluding prevalent users of loop diuretics, we minimized depletion of susceptibles, that is, a form of selection bias that can distort associations, particularly for acute outcomes.^20^

Several limitations merit consideration. First, HF diagnoses were identified based on diagnostic codes and self‐reported information, which lack detailed phenotypic granularity. Therefore, a distinction between HF with preserved or reduced ejection fraction or between acute or chronic HF, or a stratification by disease severity, was not possible. However, the overall use of guideline‐directed, disease‐modifying HF therapies was balanced across genotype groups; therefore, we deem it unlikely that differences in background medical treatment materially influenced the observed associations. Second, misclassification of loop diuretic exposure is possible; however, analyses restricted to shorter follow‐up periods yielded consistent results. Third, misclassification of the outcome may also have occurred, although stricter outcome definitions did not materially alter effect estimates. Fourth, more than 90% of patients received furosemide, while no patients were treated with torsemide. The latter has been proposed to offer pharmacokinetic advantages, including greater bioavailability and longer half‐life, and earlier observational studies suggested potential clinical benefits.^4^, ^16^, ^21^ However, the large pragmatic TRANSFORM‐HF trial (*n* = 2,859) demonstrated no difference in all‐cause mortality between torsemide and furosemide (HR 1.02, 95% CI 0.89 to 1.18).^22^ Results were consistent across subgroups and secondary outcomes in this trial. Although interpretation was limited by crossover and nonadherence, these findings suggest that major differences in hard clinical outcomes between loop diuretics in hospitalized HF are modest. Nonetheless, pharmacologic differences at the tubular level could theoretically interact with genetic variation in sodium handling, and intra‐class heterogeneity in genotype‐drug interactions applying to loop diuretics cannot be entirely excluded. Finally, because the dose of loop diuretics was not consistently reported in the data, dose‐specific analyses were not feasible.

## CONCLUSION

In this large cohort of patients with HF initiating treatment with loop diuretics, *UMOD* rs13333226 was not associated with clinical effectiveness or safety outcomes. These findings do not support a clinically meaningful pharmacogenetic role for this variant in routine HF management.

## FUNDING

There was no funding for this study. A.D. is supported by the Heisenberg Program of the German Research Foundation.

## CONFLICT OF INTEREST STATEMENT

R.K. reports Honoraria for consultancy, lectures, and support for research from: AstraZeneca, Bayer, Krka, Menarini Group, Merck, ProMed, PolPharma, Recor, Servier, and Zentiva. All other authors declared no competing interests for this work.

## AUTHOR CONTRIBUTIONS

R.K. wrote the manuscript. R.K., J.B., and A.D. designed the research. P.G., J.L., and P.T. performed the research and analyzed the data.

## Supporting information


Data S1.
