# Supplementary material for: UMOD Genotype and Clinical Outcomes in Heart Failure Patients Treated with Loop Diuretics: A UK Biobank Pharmacogenetic Cohort Study
Source: Clin Pharmacol Ther. 2026 Aug 6:10.1002/cpt.70426. Online ahead of print. doi: 10.1002/cpt.70426 (PMC13445941; doi:10.1002/cpt.70426)
Supplement: Supplementary file 1 [file CPT-9999-0-s001.docx]

**Contents**

[Table S1. ICD-10 codes for HF	2](#_Toc223345569)

[Table S2. Read codes for HF	3](#_Toc223345570)

[Table S3. Risk of worsening HF associated with the AA UMOD genotype among patients with HF initiating loop diuretics (secondary analyses)	5](#_Toc223345571)

[Table S4. Risk of MACE associated with the AA UMOD genotype among patients with HF initiating loop diuretics (secondary analyses)	6](#_Toc223345572)

[Table S5. Risk of AKI associated with the AA UMOD genotype among patients with HF initiating loop diuretics (secondary analyses)	7](#_Toc223345573)

[Table S6. Risk of all-cause mortality associated with the AA UMOD genotype among patients with HF initiating loop diuretics (secondary analyses)	8](#_Toc223345574)

[Table S7. Risk of worsening HF associated with the AA UMOD genotype among patients with HF initiating loop diuretics (sensitivity analyses)	9](#_Toc223345575)

[Table S8. Risk of MACE associated with the AA UMOD genotype among patients with HF initiating loop diuretics (sensitivity analyses)	10](#_Toc223345576)

[Table S9. Risk of AKI associated with the AA UMOD genotype among patients with HF initiating loop diuretics (sensitivity analyses)	11](#_Toc223345577)

[Table S10. Risk of all-cause mortality associated with the AA UMOD genotype among patients with HF initiating loop diuretics (sensitivity analyses)	12](#_Toc223345578)

# **Table S1. ICD-10 codes for HF**

| **Type** | **Description** |
| --- | --- |
| I11.0 | Hypertensive heart disease with (congestive) heart failure |
| I13.0 | Hypertensive heart and renal disease with (congestive) heart failure |
| I13.2 | Hypertensive heart and renal disease with both (congestive) heart failure and renal failure |
| I50.0 | Congestive heart failure |
| I50.1 | Left ventricular failure |
| I50.2 | Systolic (congestive) heart failure |
| I50.3 | Diastolic (congestive) heart failure |
| I50.4 | Combined systolic and diastolic heart failure |
| I50.9 | Heart failure, unspecified |

Abbreviations: ICD-10, International Classification of Diseases, 10th Revision; HR, heart failure.

# **Table S2. Read codes for HF**

| **Read V2** | **Read V3** | **Description^*^** |
| --- | --- | --- |
| G5800 | G5800 | Acute congestive heart failure |
| G582. | G582. | Acute heart failure |
| G5810 | G5810 | Acute left ventricular failure |
| 8H2S. | XaKNW | Admit heart failure emergency |
| G5801 | G5801 | Chronic congestive heart failure |
| G5803 | G5803 | Compensated cardiac failure |
| Q48y1 | Q48y1 | Congenital cardiac failure |
| G580. | G580. | Congestive heart failure |
|  | XE0V8 | Congestive heart failure |
|  | X202l | Congestive heart failure |
| G5804 | XaO5n | Congestive heart failure due to valvular disease |
| 662T. | XaIIU | Congestive heart failure monitoring |
| G5802 | G5802 | Decompensated cardiac failure |
|  | XaMHD | Did not attend heart failure clinic |
| 9hH1. | XaMJB | Excepted from heart failure quality indicators: Informed dissent |
| 9hH0. | XaMJA | Excepted from heart failure quality indicators: Patient unsuitable |
|  | XaMJ9 | Exception reporting: heart failure quality indicators |
| 8H7o0 | XaXBj | Fast track heart failure referral for transthoracic two dimensional echocardiogram |
|  | 14A6. | H/O: heart failure |
| 8CMK. | XaXkR | Has heart failure management plan |
|  | XaBwi | H/O: Heart failure in last year |
| G58.. | G58.. | Heart failure |
|  | XaLon | Heart failure 6 month review |
| 662W. | XaIQN | Heart failure annual review |
| 8CL3. | XaLNA | Heart failure care plan discussed with patient |
|  | XaZih | Heart failure clinical pathway |
| 1O1.. | XaIpn | Heart failure confirmed |
| 679X. | XaNUf | Heart failure education |
| 8HBE. | XaIQM | Heart failure follow-up |
| 67D4. |  | Heart failure information given to patient |
| 9Or.. | XaLN6 | Heart failure monitoring administration |
| 9Or3. | XaMGs | Heart failure monitoring first letter |
| 9Or2. | XaMGo | Heart failure monitoring verbal invite |
| G58z. | XE0V9 | Heart failure NOS |
| 9Or0. | XaLN7 | Heart failure review completed |
| G583. | XaWyi | Heart failure with normal ejection fraction |
| G581. | X102Y | Left ventricular failure |
|  | XM1Qn | Left ventricular failure |
|  | XE2QG | Left ventricular failure |
|  | G21z1 | Hypertensive heart disease NOS with congestive cardiac failure |
| 388D. | XaIL7 | New York Heart Association classification of heart failure symptoms |
|  | XabM9 | On optimal heart failure therapy |
| 8HTL. | XaILD | Referral to heart failure clinic |
| 8IE0. | XaXIR | Referral to heart failure education group declined |
| 8HHz. | XaLMx | Referral to heart failure exercise programme |
| 8HHb. | XaKNX | Referral to heart failure nurse |
| 8HTL0 | XaaH6 | Referral to rapid access heart failure clinic |
| G584. | X202l | Right ventricular failure |
| 9N2p. | XaKNa | Seen by community heart failure nurse |
| 9N0k. | XaKNN | Seen in heart failure clinic |

Abbreviations: HF, heart failure; H/O, history of; NOS, not otherwise specified.

^*^ In addition, we also considered the term ‘heart failure/pulmonary oedema’ from self-reported data.

# **Table S3. Risk of worsening HF associated with the AA UMOD genotype among patients with HF initiating loop diuretics (secondary analyses)**

|  | **N**  **Patients** | **N**  **Events** | **N**  **Person-years** | **IR (95%CI) per 100 person-years** | **Crude HR**  **(95%CI)** | **Adjusted HR^1^**  **(95%CI)** |
| --- | --- | --- | --- | --- | --- | --- |
| Age ≥65 years |  |  |  |  |  |  |
| AA | 709 | 157 | 590 | 26.6 (22.5–30.8) | 0.89 (0.68–1.15) | 0.88 (0.68–1.15) |
| AG/GG | 358 | 88 | 293 | 30.0 (23.9–36.5) | 1.00 (reference) | 1.00 (reference) |
| Age <65 years |  |  |  |  |  |  |
| AA | 239 | 61 | 190 | 32.0 (24.2–40.4) | 1.29 (0.81–2.05) | 1.25 (0.79–2.00) |
| AG/GG | 120 | 25 | 102 | 24.5 (15.7–34.3) | 1.00 (reference) | 1.00 (reference) |
| Female sex |  |  |  |  |  |  |
| AA | 321 | 71 | 269 | 26.4 (20.4–32.7) | 0.83 (0.57–1.22) | 0.79 (0.54–1.15) |
| AG/GG | 165 | 43 | 136 | 31.7 (22.8–41.2) | 1.00 (reference) | 1.00 (reference) |
| Male sex |  |  |  |  |  |  |
| AA | 627 | 147 | 512 | 28.7 (24.2–33.4) | 1.06 (0.80–1.41) | 1.05 (0.79–1.40) |
| AG/GG | 313 | 70 | 259 | 27.0 (20.8–33.6) | 1.00 (reference) | 1.00 (reference) |
| No chronic kidney disease |  |  |  |  |  |  |
| AA | 678 | 156 | 555 | 28.1 (23.8–32.6) | 1.02 (0.78–1.33) | 1.00 (0.77–1.31) |
| AG/GG | 378 | 86 | 312 | 27.5 (21.8–33.6) | 1.00 (reference) | 1.00 (reference) |
| Chronic kidney disease |  |  |  |  |  |  |
| AA | 270 | 62 | 226 | 27.5 (20.8–34.5) | 0.84 (0.53–1.32) | 0.83 (0.53–1.32) |
| AG/GG | 100 | 27 | 82 | 32.7 (20.6–46.1) | 1.00 (reference) | 1.00 (reference) |

Abbreviations: HF, heart failure; UMOD, uromodulin; IR, incidence rate; HR, hazard ratio; CI, confidence interval; BMI, body mass index.

^1^ Adjusted for age, sex, BMI, and smoking status. Participants with missing BMI (n = 6) were suppressed.

# **Table S4. Risk of MACE associated with the AA UMOD genotype among patients with HF initiating loop diuretics (secondary analyses)**

|  | **N**  **Patients** | **N**  **Events** | **N**  **Person-years** | **IR (95%CI) per 100 person-years** | **Crude HR**  **(95%CI)** | **Adjusted HR^1^**  **(95%CI)** |
| --- | --- | --- | --- | --- | --- | --- |
| Age ≥65 years |  |  |  |  |  |  |
| AA | 709 | 42 | 667 | 6.3 (4.5–8.3) | 0.84 (0.51–1.38) | 0.82 (0.50–1.34) |
| AG/GG | 358 | 25 | 334 | 7.5 (4.8–10.5) | 1.00 (reference) | 1.00 (reference) |
| Age <65 years |  |  |  |  |  |  |
| AA | 239 | 15 | 222 | 6.7 (3.6–10.3) | 1.90 (0.63–5.73) | 1.74 (0.57–5.28) |
| AG/GG | 120 | 4 | 114 | 3.5 (0.9–7.0) | 1.00 (reference) | 1.00 (reference) |
| Female sex |  |  |  |  |  |  |
| AA | 321 | 15 | 304 | 4.9 (2.6–7.6) | 1.11 (0.45–2.71) | 1.18 (0.46–2.98) |
| AG/GG | 165 | 7 | 158 | 4.4 (1.3–8.2) | 1.00 (reference) | 1.00 (reference) |
| Male sex |  |  |  |  |  |  |
| AA | 627 | 42 | 585 | 7.2 (5.1–9.4) | 0.95 (0.57–1.59) | 0.93 (0.55–1.56) |
| AG/GG | 313 | 22 | 290 | 7.6 (4.5–11.0) | 1.00 (reference) | 1.00 (reference) |
| No chronic kidney disease |  |  |  |  |  |  |
| AA | 678 | 31 | 641 | 4.8 (3.3–6.5) | 0.71 (0.42–1.22) | 0.69 (0.40–1.18) |
| AG/GG | 378 | 24 | 354 | 6.8 (4.2–9.6) | 1.00 (reference) | 1.00 (reference) |
| Chronic kidney disease |  |  |  |  |  |  |
| AA | 270 | 26 | 247 | 10.5 (6.9–14.6) | 1.95 (0.75–5.09) | 2.00 (0.76–5.27) |
| AG/GG | 100 | 5 | 94 | 5.3 (1.1–10.6) | 1.00 (reference) | 1.00 (reference) |

Abbreviations: MACE, major adverse cardiovascular events; UMOD, uromodulin; HF, heart failure; IR, incidence rate; HR, hazard ratio; CI, confidence interval; BMI, body mass index.

^1^ Adjusted for age, sex, BMI, and smoking status. Participants with missing BMI (n = 6) were suppressed.

# **Table S5. Risk of AKI associated with the AA UMOD genotype among patients with HF initiating loop diuretics (secondary analyses)**

|  | **N**  **Patients** | **N**  **Events** | **N**  **Person-years** | **IR (95%CI) per 100 person-years** | **Crude HR**  **(95%CI)** | **Adjusted HR^1^**  **(95%CI)** |
| --- | --- | --- | --- | --- | --- | --- |
| Age ≥65 years |  |  |  |  |  |  |
| AA | 709 | 49 | 659 | 7.4 (5.5–9.6) | 0.98 (0.60–1.58) | 0.95 (0.59–1.54) |
| AG/GG | 358 | 25 | 328 | 7.6 (4.9–10.7) | 1.00 (reference) | 1.00 (reference) |
| Age <65 years |  |  |  |  |  |  |
| AA | 239 | 13 | 223 | 5.8 (2.7–9.4) | 1.67 (0.54–5.11) | 1.65 (0.54–5.09) |
| AG/GG | 120 | 4 | 115 | 3.5 (0.9–7.0) | 1.00 (reference) | 1.00 (reference) |
| Female sex |  |  |  |  |  |  |
| AA | 321 | 17 | 303 | 5.6 (3.0–8.6) | 1.25 (0.52–3.02) | 1.31 (0.54–3.16) |
| AG/GG | 165 | 7 | 157 | 4.5 (1.3–8.3) | 1.00 (reference) | 1.00 (reference) |
| Male sex |  |  |  |  |  |  |
| AA | 627 | 45 | 579 | 7.8 (5.5–10.2) | 1.01 (0.61–1.68) | 0.99 (0.59–1.66) |
| AG/GG | 313 | 22 | 286 | 7.7 (4.5–11.2) | 1.00 (reference) | 1.00 (reference) |
| No chronic kidney disease |  |  |  |  |  |  |
| AA | 678 | 34 | 637 | 5.3 (3.6–7.2) | 1.05 (0.59–1.85) | 1.04 (0.58–1.84) |
| AG/GG | 378 | 18 | 352 | 5.1 (2.8–7.7) | 1.00 (reference) | 1.00 (reference) |
| Chronic kidney disease |  |  |  |  |  |  |
| AA | 270 | 28 | 245 | 11.4 (7.3–15.9) | 0.95 (0.47–1.90) | 0.90 (0.44–1.82) |
| AG/GG | 100 | 11 | 91 | 12.1 (5.5–19.8) | 1.00 (reference) | 1.00 (reference) |

Abbreviations: AKI, acute kidney injury; UMOD, uromodulin; HF, heart failure; IR, incidence rate; HR, hazard ratio; CI, confidence interval; BMI, body mass index.

^1^ Adjusted for age, sex, BMI, and smoking status. Participants with missing BMI (n = 6) were suppressed.

# **Table S6. Risk of all-cause mortality associated with the AA UMOD genotype among patients with HF initiating loop diuretics (secondary analyses)**

|  | **N**  **Patients** | **N**  **Events** | **N**  **Person-years** | **IR (95%CI) per 100 person-years** | **Crude HR**  **(95%CI)** | **Adjusted HR^1^**  **(95%CI)** |
| --- | --- | --- | --- | --- | --- | --- |
| Age ≥65 years |  |  |  |  |  |  |
| AA | 709 | 53 | 676 | 7.8 (5.8–10.1) | 1.03 (0.65–1.65) | 1.02 (0.63–1.63) |
| AG/GG | 358 | 26 | 343 | 7.6 (5.0–10.5) | 1.00 (reference) | 1.00 (reference) |
| Age <65 years |  |  |  |  |  |  |
| AA | 239 | 20 | 228 | 8.8 (5.3–12.7) | 2.03 (0.76–5.42) | 1.99 (0.75–5.30) |
| AG/GG | 120 | 5 | 116 | 4.3 (0.9–8.6) | 1.00 (reference) | 1.00 (reference) |
| Female sex |  |  |  |  |  |  |
| AA | 321 | 20 | 310 | 6.4 (3.9–9.3) | 1.29 (0.57–2.93) | 1.35 (0.59–3.09) |
| AG/GG | 165 | 8 | 160 | 5.0 (1.9–8.7) | 1.00 (reference) | 1.00 (reference) |
| Male sex |  |  |  |  |  |  |
| AA | 627 | 53 | 594 | 8.9 (6.6–11.5) | 1.16 (0.71–1.89) | 1.14 (0.70–1.86) |
| AG/GG | 313 | 23 | 299 | 7.7 (4.7–11.0) | 1.00 (reference) | 1.00 (reference) |
| No chronic kidney disease |  |  |  |  |  |  |
| AA | 678 | 44 | 651 | 6.8 (4.8–8.8) | 1.03 (0.62–1.69) | 1.02 (0.62–1.68) |
| AG/GG | 378 | 24 | 364 | 6.6 (4.1–9.3) | 1.00 (reference) | 1.00 (reference) |
| Chronic kidney disease |  |  |  |  |  |  |
| AA | 270 | 29 | 253 | 11.4 (7.5–15.8) | 1.55 (0.68–3.54) | 1.53 (0.66–3.53) |
| AG/GG | 100 | 7 | 95 | 7.3 (2.1–13.6) | 1.00 (reference) | 1.00 (reference) |

Abbreviations: HF, heart failure; UMOD, uromodulin; IR, incidence rate; HR, hazard ratio; CI, confidence interval; BMI, body mass index.

^1^ Adjusted for age, sex, BMI, and smoking status. Participants with missing BMI (n = 6) were suppressed.

# **Table S7. Risk of worsening HF associated with the AA UMOD genotype among patients with HF initiating loop diuretics (sensitivity analyses)**

|  | **N**  **Patients** | **N**  **Events** | **N**  **Person-years** | **IR (95%CI) per 100 person-years** | **Crude HR**  **(95%CI)** | **Adjusted HR^1^**  **(95%CI)** |
| --- | --- | --- | --- | --- | --- | --- |
| European ancestry only |  |  |  |  |  |  |
| AA | 819 | 186 | 676 | 27.5 (23.7–31.5) | 0.96 (0.75–1.23) | 0.94 (0.73–1.21) |
| AG/GG | 406 | 96 | 336 | 28.6 (22.9–34.5) | 1.00 (reference) | 1.00 (reference) |
| 3-month follow-up |  |  |  |  |  |  |
| AA | 948 | 101 | 217 | 46.6 (37.8–55.8) | 0.94 (0.67–1.31) | 0.91 (0.65–1.27) |
| AG/GG | 478 | 54 | 109 | 49.7 (36.8–63.5) | 1.00 (reference) | 1.00 (reference) |
| 6-month follow-up |  |  |  |  |  |  |
| AA | 948 | 156 | 419 | 37.2 (31.5–43.2) | 1.06 (0.80–1.40) | 1.04 (0.78–1.37) |
| AG/GG | 478 | 74 | 211 | 35.0 (27.4–43.1) | 1.00 (reference) | 1.00 (reference) |
| Stricter outcome definition |  |  |  |  |  |  |
| AA | 948 | 69 | 867 | 8.0 (6.1–9.9) | 0.94 (0.63–1.40) | 0.89 (0.60–1.33) |
| AG/GG | 480 | 37 | 439 | 8.4 (5.9–11.2) | 1.00 (reference) | 1.00 (reference) |

Abbreviations: HF, heart failure; UMOD, uromodulin; IR, incidence rate; HR, hazard ratio; CI, confidence interval; BMI, body mass index.

^1^ Adjusted for age, sex, BMI, and smoking status. Participants with missing BMI (n = 6) were suppressed.

# **Table S8. Risk of MACE associated with the AA UMOD genotype among patients with HF initiating loop diuretics (sensitivity analyses)**

|  | **N**  **Patients** | **N**  **Events** | **N**  **Person-years** | **IR (95%CI) per 100 person-years** | **Crude HR**  **(95%CI)** | **Adjusted HR^1^**  **(95%CI)** |
| --- | --- | --- | --- | --- | --- | --- |
| European ancestry only |  |  |  |  |  |  |
| AA | 819 | 42 | 770 | 5.5 (3.9–7.1) | 0.77 (0.47–1.24) | 0.75 (0.46–1.22) |
| AG/GG | 406 | 27 | 379 | 7.1 (4.5–10.0) | 1.00 (reference) | 1.00 (reference) |
| 3-month follow-up |  |  |  |  |  |  |
| AA | 948 | 19 | 229 | 8.3 (4.8–12.2) | 0.68 (0.34–1.36) | 0.71 (0.35–1.42) |
| AG/GG | 478 | 14 | 115 | 12.2 (6.1–19.1) | 1.00 (reference) | 1.00 (reference) |
| 6-month follow-up |  |  |  |  |  |  |
| AA | 948 | 40 | 458 | 8.7 (6.1–11.6) | 0.91 (0.54–1.54) | 0.89 (0.53–1.51) |
| AG/GG | 478 | 22 | 230 | 9.6 (5.7–13.9) | 1.00 (reference) | 1.00 (reference) |
| Stricter outcome definition |  |  |  |  |  |  |
| AA | 948 | 51 | 893 | 5.7 (4.3–7.3) | 0.95 (0.60–1.52) | 0.94 (0.58–1.50) |
| AG/GG | 478 | 27 | 450 | 6.0 (3.8–8.5) | 1.00 (reference) | 1.00 (reference) |

Abbreviations: MACE, major adverse cardiovascular events; UMOD, uromodulin; HF, heart failure; IR, incidence rate; HR, hazard ratio; CI, confidence interval; BMI, body mass index.

^1^ Adjusted for age, sex, BMI, and smoking status. Participants with missing BMI (n = 6) were suppressed.

# **Table S9. Risk of AKI associated with the AA UMOD genotype among patients with HF initiating loop diuretics (sensitivity analyses)**

|  | **N**  **Patients** | **N**  **Events** | **N**  **Person-years** | **IR (95%CI) per 100 person-years** | **Crude HR**  **(95%CI)** | **Adjusted HR^1^**  **(95%CI)** |
| --- | --- | --- | --- | --- | --- | --- |
| European ancestry only |  |  |  |  |  |  |
| AA | 819 | 56 | 763 | 7.3 (5.5–9.3) | 1.15 (0.71–1.86) | 1.14 (0.70–1.84) |
| AG/GG | 406 | 24 | 376 | 6.4 (4.0–9.0) | 1.00 (reference) | 1.00 (reference) |
| 3-month follow-up |  |  |  |  |  |  |
| AA | 948 | 19 | 228 | 8.3 (4.8–12.3) | 0.73 (0.36–1.49) | 0.74 (0.36–1.50) |
| AG/GG | 478 | 13 | 115 | 11.3 (5.2–18.3) | 1.00 (reference) | 1.00 (reference) |
| 6-month follow-up |  |  |  |  |  |  |
| AA | 948 | 39 | 456 | 8.6 (5.9–11.4) | 0.98 (0.57–1.68) | 0.96 (0.56–1.66) |
| AG/GG | 478 | 20 | 228 | 8.8 (5.3–12.7) | 1.00 (reference) | 1.00 (reference) |
| Stricter outcome definition |  |  |  |  |  |  |
| AA | 948 | 12 | 899 | 1.3 (0.7–2.1) | 0.67 (0.28–1.60) | 0.71 (0.30–1.70) |
| AG/GG | 478 | 9 | 455 | 2.0 (0.9–3.3) | 1.00 (reference) | 1.00 (reference) |

Abbreviations: AKI, acute kidney injury; UMOD, uromodulin; HF, heart failure; IR, incidence rate; HR, hazard ratio; CI, confidence interval; BMI, body mass index.

^1^ Adjusted for age, sex, BMI, and smoking status. Participants with missing BMI (n = 6) were suppressed.

# **Table S10. Risk of all-cause mortality associated with the AA UMOD genotype among patients with HF initiating loop diuretics (sensitivity analyses)**

|  | **N**  **Patients** | **N**  **Events** | **N**  **Person-years** | **IR (95%CI) per 100 person-years** | **Crude HR**  **(95%CI)** | **Adjusted HR^1^**  **(95%CI)** |
| --- | --- | --- | --- | --- | --- | --- |
| European ancestry only |  |  |  |  |  |  |
| AA | 819 | 59 | 784 | 7.5 (5.6–9.6) | 1.09 (0.69–1.71) | 1.08 (0.68–1.71) |
| AG/GG | 406 | 27 | 390 | 6.9 (4.4–9.7) | 1.00 (reference) | 1.00 (reference) |
| 3-month follow-up |  |  |  |  |  |  |
| AA | 948 | 28 | 230 | 12.2 (7.8–16.9) | 1.28 (0.64–2.58) | 1.25 (0.62–2.53) |
| AG/GG | 478 | 11 | 116 | 9.5 (4.3–15.5) | 1.00 (reference) | 1.00 (reference) |
| 6-month follow-up |  |  |  |  |  |  |
| AA | 948 | 48 | 462 | 10.4 (7.6–13.4) | 1.21 (0.72–2.04) | 1.19 (0.70–2.01) |
| AG/GG | 478 | 20 | 234 | 8.6 (5.1–12.4) | 1.00 (reference) | 1.00 (reference) |

Abbreviations: UMOD, uromodulin; HF, heart failure; IR, incidence rate; HR, hazard ratio; CI, confidence interval; BMI, body mass index.

^1^ Adjusted for age, sex, BMI, and smoking status. Participants with missing BMI (n = 6) were suppressed.
